# Low-Density Lipoprotein Modified by Myeloperoxidase in Inflammatory Pathways and Clinical Studies

**DOI:** 10.1155/2013/971579

**Published:** 2013-07-24

**Authors:** Cédric Delporte, Pierre Van Antwerpen, Luc Vanhamme, Thierry Roumeguère, Karim Zouaoui Boudjeltia

**Affiliations:** ^1^Laboratory of Pharmaceutical Chemistry, Faculty of Pharmacy, Université Libre de Bruxelles, Boulevard du Triomphe, Campus Plaine CP 205/5, 1050 Brussels, Belgium; ^2^Analytical Platform of the Faculty of Pharmacy, Université Libre de Bruxelles, Boulevard du Triomphe, Campus Plaine CP 205/5, 1050 Brussels, Belgium; ^3^Institute for Molecular Biology and Medicine (IBMM), Université Libre de Bruxelles, Rue des Professeurs Jeener et Brachet 12, 6041 Gosselies, Belgium; ^4^Department of Urology, Erasme University Hospital, Université Libre de Bruxelles, Route de Lennik 808, 1070 Brussels, Belgium; ^5^Laboratory of Experimental Medicine (ULB 222 Unit), CHU de Charleroi, A. Vésale Hospital, Université Libre de Bruxelles, Rue de Gozée 706, 6110 Montigny-le-Tilleul, Belgium

## Abstract

Oxidation of low-density lipoprotein (LDL) has a key role in atherogenesis. Among the different models of oxidation that have been studied, the one using myeloperoxidase (MPO) is thought to be more physiopathologically relevant. Apolipoprotein B-100 is the unique protein of LDL and is the major target of MPO. Furthermore, MPO rapidly adsorbs at the surface of LDL, promoting oxidation of amino acid residues and formation of oxidized lipoproteins that are commonly named Mox-LDL. The latter is not recognized by the LDL receptor and is accumulated by macrophages. In the context of atherogenesis, Mox-LDL accumulates in macrophages leading to foam cell formation. Furthermore, Mox-LDL seems to have specific effects and triggers inflammation. Indeed, those oxidized lipoproteins activate endothelial cells and monocytes/macrophages and induce proinflammatory molecules such as TNF**α** and IL-8. Mox-LDL may also inhibit fibrinolysis mediated via endothelial cells and consecutively increase the risk of thrombus formation. Finally, Mox-LDL has been involved in the physiopathology of several diseases linked to atherosclerosis such as kidney failure and consequent hemodialysis therapy, erectile dysfunction, and sleep restriction. All these issues show that the investigations of MPO-dependent LDL oxidation are of importance to better understand the inflammatory context of atherosclerosis.

## 1. Introduction

Atherosclerosis is an inflammatory process involving vascular cells, monocytes, T lymphocytes, proinflammatory cytokines, chemoattractant cytokines (chemokines), and growth factors [1–3]. Specific arterial regions are favorable to atherosclerosis development [4], and these areas have been linked to shear stress abnormalities [5]. More recently, it was shown in apoE−/− mice that smooth muscle cells display a different transcriptome at locations where atherogenesis is prone even before the development of the lesion [6]. The accumulation of foam cells in intima leads to primary lesions characterized by fatty streaks in the artery wall and by thickening of the wall. Early lesions are found in the aorta of healthy 10-year-old children, in coronary arteries of 20-year-old adults, and later in cerebral arteries [7]. These lesions can naturally disappear without causing any disorder to the patient or progress of advanced lesions with smooth muscle cell migration and proliferation, foam cell accumulation, and can even lead to plaque rupture and thrombus formation.

Among the factors associated with this process, modification and particularly oxidation of low-density lipoproteins (LDLs) have been of major interest since Steinberg et al. showed that native LDL does not accumulate in macrophages, whereas modified lipoprotein does [8, 9]. However, the exact mechanisms of LDL oxidation are still not completely understood, and researchers continue to argue about them [10]. Several mechanisms have been described including reactive oxygen species (ROS) produced by endothelial cells and monocytes/macrophages [11], metal ions [12], lipoxygenase [13], or myeloperoxidase [14, 15]. Each oxidative mechanism of lipoprotein is characterized by targeting either lipid, protein, or both moieties [8].

Highly oxidized LDL (ox-LDL) cannot bind to the LDL receptor and is taken up by monocytes which transform into macrophages. Indeed, these cells express scavenger receptors such as (SR) such as CD36, SR-A, SR-B1, and LOX-1 at their surface, which bind ox-LDL and enable scavenger receptor-mediated endocytosis [16]. This reaction is the best way for removing excess of ox-LDL in the arterial wall. Conversely, this process could worsen, and ox-LDL continues to accumulate in the subendothelial space. Macrophages continue to engulf the modified lipoproteins and evolve to a state where high quantities of lipids are intracellularly accumulated leading to foam cell formation [17]. Resistance of ox-LDL to acidic lysosomal proteolysis via cathepsins has also been observed [18]. The latter phenomenon increases the risk of LDL accumulation in macrophages and therefore foam cell formation. Foam cells themselves have a proinflammatory effect by producing cytokines and growth factors such as interleukins (IL) 1*β* and -8, interferon-*γ*, tumor-necrosis factor-*α* (TNF*α*), and macrophage colony stimulating factor (M-CSF).

Ox-LDL is widely described as a key component of atherogenesis and triggers the inflammatory processes of the disease. Ox-LDL induces a number of potentially proatherogenic activities such as the production of proinflammatory cytokines and chemokines by monocytes, endothelial cells, and smooth muscle cells *in vitro* [19, 20]. In this paper, we focused on a particular and frequent LDL oxidation mechanism involving myeloperoxidase (MPO). MPO is an important enzyme of neutrophils which combat pathogen invasion in the body. Indeed, MPO catalyzes the production of oxidative reagents which damage pathogens and aid in their elimination. Unfortunately, in chronic inflammation syndromes, MPO is also released into the extracellular space due to neutrophil activation where MPO-derived oxidants can in turn cause tissue damage. One of the targeted components is LDL, leading to MPO-dependent oxidized LDL, commonly named Mox-LDL. 

In this paper, we first review LDL, apolipoprotein B-100, the unique protein of LDL, and its oxidation sensitive components. MPO and its enzymatic mechanism are then briefly described. Following this, modifications of LDL are discussed with particular focus on MPO-dependent oxidation mechanisms and the specificity of MPO to modify LDL. *In vitro* experiments on inflammation involving Mox-LDL are then addressed. In this section, we will show that Mox-LDL has a key role in triggering the inflammatory response during atherogenesis and has effects on monocytes, macrophages, and endothelial cells and that those effects are different than LDL modified by other systems. Finally, clinical aspects of Mox-LDL are illustrated, focusing on several conditions such as atherosclerosis, erectile dysfunction, dialysis, nonalcoholic fatty liver disease, and sleep disorders.

## 2. Low-Density Lipoprotein and Apolipoprotein B-100

LDL is one of the major carriers of cholesterol in the human body and plays a role in cholesterol metabolism, as well as other lipoproteins such as high-density, intermediate-density, or very-low-density lipoproteins. LDL is generally considered to be a spherical particle of about 22 nm in diameter [21]. It includes two major groups of compounds: (i) lipids and (ii) protein representing 80% and 20% of total lipoprotein weight, respectively. 

The lipid moiety contains approximately 3000 molecules including cholesterol esters mainly but also free cholesterol, phospholipids, and triglycerides. In LDL, lipids are separated into two parts: (i) a monolayer of phospholipids and free cholesterol at the surface and (ii) a core majorly composed of cholesterol esters but also free cholesterol and triglycerides. 

The protein moiety of LDL includes a unique protein which is an exception among lipoproteins. This protein is apolipoprotein B-100 (apoB-100) and was completely sequenced for the first time in 1986 by several labs thanks to genetic investigations [22–25]. Mature apoB-100 consists of 4536 amino acid residues with 19 putative N-glycosylation sites making it one of the largest monomeric proteins in the human body with a molecular mass estimated to be 550 kDa. ApoB-100 is usually divided into 5 domains as first described by Yang et al. in 1989 [26] and later summarized by Segrest et al. [27]. This division of apoB-100 follows *α*- and *β*-domain characteristics. The consensus structure is as follows: NH_2_-*βα*1-*β*1-*α*2-*β*2-*α*3-COOH. ApoB-100 is distributed both at the surface and in the core of the lipoprotein where the NH_2_-*βα*1 domain (the first 1000 residues) is generally described as being a highly hydrophilic domain located outside the particle. Although the latter domain is hydrophilic, apoB-100 has several hydrophobic segments which enable strong interactions with lipids (core and surface lipids) and stabilize the lipoprotein structure.

ApoB-100 is also a key player in LDL recognition and binding to the LDL receptor, which is present at the surface of most human cells. When LDL binds to the receptor, uptake occurs followed by degradation of the lipoprotein and release of cholesterol for the cell's needs. Modifications to the apoB-100 structure can lead to decrease in affinity or even the inability of LDL to bind the receptor [28, 29]. More than 50 variants are currently described in UniProt Database, many with no effect on LDL function although others have harmful properties resulting in hypercholesterolemia and its deleterious effects. It is also assumed that a receptor-binding site is present at the surface of apoB-100. Many studies have tried to determine the exact binding site, but it is still controversial [22, 30–33]. The sequence between residues 3345 and 3381 would include the receptor-binding site and sometimes the 3359–3369 segment is mentioned [28], but it should be kept in mind that mutations on apoB-100 close to this binding site also disrupt the ability of LDL to bind to LDL receptor (e.g., R3500Q mutation which is the major cause of familial defective apoB-100 disease). Finally, oxidized apoB-100 also has a decreased affinity for the LDL receptor. 

The characteristics of apoB-100 make its analysis a very complex and difficult task. Since its sequencing in 1986, apoB-100 has been the subject of several studies to elucidate its exact structure [27, 34–37]. In 1989, Yang et al. used a specific methodology to distinguish segments of the protein which are hydrophobic from those which are hydrophilic [38]. This approach is useful for prediction and supposition of segments which could be more sensitive to post-translational modifications (PTMs). 

The best experimental procedures to study the modifications that occur to apoB-100 are those that utilize mass spectrometers (MS/MS or MS^*n*^) [39]. Indeed, many of these instruments have a high-resolution mass analyzer coupled to the ability to fragment the peptides in order to sequence them. Such instruments include quadrupole-time of flight (QToF) mass spectrometers which can detect peptide and PTMs with high accuracy which can be coupled to separation techniques such as liquid chromatography. This represents a powerful strategy to discover PTMs on proteins. 

Another strategy has been recently described to analyze modifications of apoB-100 taking advantage of known modifications and their specific product ions to monitor PTMs by LC-MS/MS [40]. However, the huge sequence of apoB-100 also makes it important to optimize all parameters of the analysis. For this purpose, we developed and optimized an LC-MS/MS method capable of recovering up to 80% of apoB-100, and we have shown that this is required to detect the maximum of PTMs currently achievable (4 times more modifications were recovered thanks to the optimized protocol) [41].

In summary, LDL and apoB-100 investigations are important to understand their implications in the processes of disease. However, LDL/apoB-100 complexity make these studies particularly difficult, but recent improvements in instrumentation, such as those for mass spectrometry, are very helpful. In the following paragraphs, we principally discuss MPO-dependent oxidation of LDL and its roles in inflammation *in vitro* as well as *in vivo*.

## 3. Myeloperoxidase and MPO/H_2_O_2_/Halide System

MPO is a key enzyme in innate immunity and defense against pathogens [42]. Hereinafter, we will describe the major points of interest of MPO devoted to LDL oxidation and inflammation. For reviews on molecular mechanisms as well as physiological and physiopathological aspects of MPO, see Klebanoff [43], Davies [44], Davies et al. [45], and van der Veen et al. [46]. MPO expression is limited to myeloid cells, and its synthesis in neutrophils starts at the promyelocyte stage and terminates at the beginning of the myelocyte stage. Mature MPO is packed in azurophilic granules of neutrophils and accounts for 5% of the total dry cell weight, making MPO the major protein of neutrophils. It is also present in monocytes but to a lesser extent [47, 48].

MPO is a hemeglycoprotein with a mass of 140–155 kDa [49]. Its biosynthesis is a complex process including proteolytic events, heme and glycan additions, and a final dimerization step [50, 51]. Briefly, nascent MPO, called preproMPO, undergoes a first proteolytic event and N-glycan addition to make apoproMPO in the endoplasmic reticulum. The latter lacks the hememoiety which is then inserted due to the activity of chaperones (calreticulin and calnexin) which interact with MPO oligosaccharides. This forms proMPO which leaves the endoplasmic reticulum and travels to the Golgi apparatus and granules where MPO undergoes several new proteolytic events. The final monomer of MPO consists of a light chain of 106 residues and a heavy chain of 467 residues. The two chains are linked by a disulfide bond and also via the heme group. In the mature form, MPO is a dimer of two monomers linked by a disulfide bond on position cysteine 369 of each heavy chain. Each monomer is enzymatically active and can produce oxidants. MPO also contains a calcium binding site contributing to the stabilization of the structure. N-glycans play a key role in protein synthesis and also in enzymatic activity as recently shown by our experiments [52]. Furthermore, MPO is a highly cationic protein with a pI *≈* 11 enabling its binding to electronegative surfaces such as endothelial wall, lipoprotein, or proteoglycans [53, 54]. 

In the azurophilic granules, MPO is kept in an inactive state as long as the neutrophil is not activated and hydrogen peroxide (H_2_O_2_) is absent [43]. After phagocytosis, activation of neutrophils leads to the release of the contents of the azurophilic granules (including MPO) into phagosomes and to the assembly of the NADPH oxidase enzyme complex (NOX_2_) that produces superoxide radicals (O_2_^•−^). This radical is highly reactive and unstable and is rapidly converted into H_2_O_2_ spontaneously or by the action of superoxide dismutase. H_2_O_2_, which has a lower oxidation potential, can reach the ingested pathogen and contributes to its destruction by oxidizing vital molecules [55]. However, the reactivity of H_2_O_2_ alone does not produce optimal antimicrobial efficacy.

**Figure 1:**
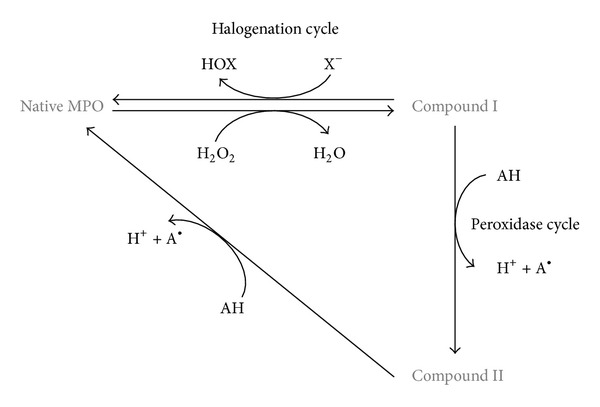
Scheme of the interconversion between different oxidized states of myeloperoxidase. The first reaction is the oxidation of native MPO to Compound I by a two-electron reaction. In the halogenation cycle, Compound I is backconverted to native MPO, and a two-electron oxidation of (pseudo-) halide generates hypo- (pseudo-) halogenous acid. In the peroxidase cycle, Compound I can oxidize an electron donor via 1-electron process transforming Compound I to Compound II and the electron donor to a radical product. Compound II can be reduced to native MPO by using 1 electron from other electron donors.

Using H_2_O_2_ and chloride ion (Cl^−^), MPO produces a more powerful oxidant molecule, namely, hypochlorous acid (HOCl). MPO can also use other (pseudo-) halide anions including Br^−^, I^−^, and SCN^−^ to give the corresponding hypo- (pseudo-) halogenous oxidants (Figure 1). The first reaction of MPO is its oxidation by H_2_O_2_ to give Compound I. In the halogenation cycle, MPO is then reduced back to its native form in a two-electron reaction. The latter enables the generation of hypo- (pseudo-) halogenous acid. Although Cl^−^ has the lowest reactivity to MPO among (pseudo-) halide anions [56], it is considered to be the major physiological substrate of MPO due to its high *in vivo* concentration [57–60]. HOCl is a strong oxidant, and it is thought to be more efficient than H_2_O_2_ in killing pathogens [61]. HOCl effectively attacks biomolecules of the ingested pathogen resulting in the death of pathogen in the phagosome. 

It is worth noting that MPO also has a peroxidase cycle in which electron donors can be oxidized and native MPO is regenerated in a two-step reaction (via the formation of Compound II: see Figure 1).

Due to its powerful oxidation products, MPO would be required to give the neutrophil optimum antimicrobial activity. Although neutrophils retain normal phagocytosis activity when MPO is inhibited or deficient, they cannot kill all types of ingested pathogens [62].

Despite its key role in host defense, MPO has also been involved in pathologic states. Indeed, during chronic inflammation or acute oxidative stress, MPO is released into the extracellular space where oxidants can be produced and host tissues damaged. Among biomolecular targets of MPO, LDL has been pointed out, and MPO is considered to be a major contributor of ox-LDL generation *in vivo* [8]. Moreover, clinical studies have highlighted serum MPO levels as a prognosis factor in patients with acute coronary syndromes or chest pain. These data support the necessity to understand the *in vivo* impact of Mox-LDL [63, 64], resulting from the reaction of MPO in the presence of LDL. 

## 4. Modification of LDL, Myeloperoxidase, and Mox-LDL

### 4.1. Introduction

One of the primary steps of atherogenesis is the activation of the immune and vasculature systems leading to endothelial dysfunction and infiltration of immune cells and LDL into the vascular wall. This also leads to an oxidative burst and production of reactive oxygen species (ROS). The latter play a key role in the disease by inducing LDL modification (oxidation) resulting in the accumulation of lipids in macrophages, and the formation of foam cells and atherosclerotic plaques. LDL can be subjected to a variety of PTMs [40] among which oxidation [12, 14, 65], glycation [66, 67], and glycosylation [68, 69] are described. The oxidation mechanism is described from here on, focusing on MPO-dependent oxidation.

### 4.2. Oxidation of LDL

Increased plasma cholesterol level is a well-documented proatherogenic factor, and hypocholesterolemic therapy is the only approved pharmacological treatment. However, around 50% of patients experiencing a cardiovascular event have a normal level of cholesterol. This fact leads researchers and physicians to consider that the quality of lipoproteins might be more important than the quantity. In this context, it is largely admitted that the modifications of lipoproteins, particularly LDL and HDL, are of major importance in the development of atherosclerosis. This was first highlighted by Steinberg et al. who observed that native LDL is not extensively taken up by macrophages and does not lead to foam cell formation even though modified lipoproteins accumulate in these cells [9, 16]. Among the modifications, oxidation of LDL has certainly been the most studied for the last number of decades, and many studies have described the presence of ox-LDL in atheromatous lesions [14, 70–72]. 

Several mechanisms of oxidation exist involving metal cations as well as many different enzyme systems such as lipoxygenase, myeloperoxidase, xanthine oxidase, and NADPH oxidase. See Yoshida and Kisugi 2010 [10] for a review on major mechanisms of LDL oxidation. Numerous oxidants preferentially target the lipid moiety (i.e., Cu^2+^, lipoxygenases, and RNS; [72–74]), whereas others target the protein moiety of lipoproteins (HOCl and MPO; [59, 75]). From here on, we summarize some of the mechanisms involved in LDL oxidation, starting with an introduction to metal cation- and lipoxygenase-dependent LDL oxidation, followed by a complete description of the MPO-dependent process.

#### 4.2.1. Oxidation of LDL by Metal Cations and Lipoxygenases

The exact process of oxidation of LDL *in vivo* is controversial. Since the discovery of the impact of LDL oxidation in atherosclerosis, metal ion-dependent oxidation of LDL has been extensively used for *in vitro* experiments. Iron (Fe^3+^) and copper (Cu^2+^) are the two major metal ions described to catalyze LDL oxidation. Copper had sometimes been preferred due to its ability to bind to apoB-100 and form a complex with it [12]. However, copper cations seem to target the lipid moiety of LDL and not apoB-100 [76]. Recently, Kriško et al. studied the impact of Cu^2+^ on the apoB-100 structure and observed conformational modifications early in the oxidation process, principally in *β*-sheet regions [77]. Metal ion-dependent oxidation mechanisms assume a high concentration of cations at the site of oxidation, a subject which is controversial [10]. Nevertheless, Stadler et al. quantified both copper- and iron-free cations using a technique that did not release transition metals from proteins during the reaction mechanism [78]. Furthermore, in this study, the authors showed an increase in both copper and iron levels in the intima of lesions compared with healthy controls. In addition, they correlated the iron levels, but not copper levels, with cholesterol levels. Whereas these cations are present, their implication in atherosclerosis remains a point of contention. Indeed, studies have sometimes positively correlated metal ion levels with cardiovascular risk, whereas others have negatively correlated them [79]. Nevertheless, epidemiological studies as well as *in vitro* experiments with iron agree on its potential impact on atherogenesis, whereas copper might be ambiguous [80–82]. 

Lipoxygenase-dependent LDL oxidation is also a contentious hypothesis because they are intracellular enzymes. However, 15-lipoxygenase mRNA and protein, as well as epitopes of ox-LDL, have been colocalized in human lesions [83, 84]. Lipoxygenase could migrate from the cytoplasm to the membrane surface of macrophages where LDL could be oxidized without phagocytosis/endocytosis of the lipoprotein. Lipoxygenases would be able to promote lipid peroxidation either directly by action on LDL lipids or indirectly by triggering ROS formation and subsequent LDL oxidation [10].

Several groups, like ours, have thus focused their research on the MPO-dependent mechanism of LDL oxidation which might be more physiologically relevant than the copper-dependent oxidation of LDL.

#### 4.2.2. Oxidation of LDL by Myeloperoxidase

*Background.* The first evidence of MPO implication in atherogenesis was highlighted in 1994 by Daugherty et al. when they observed that MPO was expressed in atherosclerotic lesions [65]. Since then, many clues have arisen such as the fact that fingerprints for *in vivo* modification by the MPO/H_2_O_2_/Cl^−^ system of apoB-100 were observed by immunohistological analyses [15] and later confirmed by gas chromatography-mass spectrometry [85, 86]. Other reports also showed that MPO deficiency or low plasma levels of MPO decrease cardiovascular risk in patients [87, 88] strengthening the case that MPO is a key element for oxidative damages in atherosclerosis.

*Targets of MPO on LDL and Products of Oxidation*. It is important to keep in mind that HOCl is the most abundant product of MPO *in vivo,* and traces of HOCl-modified epitopes have been found in acute and chronic vascular inflammatory diseases such as atherosclerosis [14, 89]. MPO produces HOCl by the enzymatic system MPO/H_2_O_2_/Cl^−^. However, to facilitate the experimental scheme, HOCl reactant is commonly used instead of the enzymatic system.

Modification of LDL by HOCl has been studied by different groups, and Malle et al. have recently reviewed the effects of that reactant on LDL [90]. From these works, it clearly appeared that the protein moiety apoB-100 is the major target of HOCl although the production of hydroperoxides, chlorohydrins, chlorinated sterols or fatty acids, and lysophospholipids has also been described in the presence of HOCl [76, 91, 92]. These lipid oxidations occur in strict reaction conditions such as an acidic pH (3–5) and with a large excess of reactant.

*Specificity of MPO to Oxidize LDL*. As mentioned above, HOCl added as a reactant has been usually used to mimic the MPO/H_2_O_2_/Cl^−^ system. However, this model may not be a perfect model to mimic MPO action on LDL/apoB-100. The main reason for this is the fact that MPO rapidly adsorbs at the surface of LDL and seems to have a strong interaction with the protein moiety of LDL [93, 94]. This adsorption phenomenon is due to the cationic characteristic of MPO, and it has been described on lipoproteins and also on endothelial cells [95]. LDL-MPO bound was first shown by Carr et al. who observed a coprecipitation of apoB-100-containing lipoproteins and MPO. The authors demonstrated that lipoprotein-deficient plasma did not permit MPO precipitation, whereas a dose-dependent MPO precipitation was observed by addition of LDL [93]. More recently, Sokolov et al. studied the specificity of MPO to bind different lipoproteins and concluded that MPO binds LDL more avidly and more specifically than HDL [94]. They also demonstrated that ceruloplasmin, which is a human plasmatic protein and a physiological MPO inhibitor [96], is able to inhibit MPO activity when HDL is present but not in the presence of LDL. The same group then studied the binding site for MPO on apoB-100 [97]. The authors predicted that MPO should bind to the NH_2_-*βα*1 domain of apoB-100 because this domain is exposed on the outside of the lipoprotein. Furthermore, because of the cationic property of MPO, these authors speculated that the MPO binding site on apoB-100 may not include any positively charged residues (lysine or Arginine) but at the opposite include negatively charged ones (aspartic or glutamic acid). They therefore proposed that MPO might bind one of the three following apoB-100 sites: ^1^EEEMLEN^7^, ^53^VELEVPQ^59^, or ^445^EQIQDDCTGDED^456^. They synthesized these three peptides and studied their affinity for MPO. Only the ^445^EQIQDDCTGDED^456^ peptide was able to form a complex with MPO, and the authors concluded that it might be the binding site of MPO on apoB-100. However, experiments should be performed to confirm this binding site. Experiments with site-specific mutations on apoB-100 to disable MPO-LDL complex formation would be a good experimental procedure, in this respect and this has previously been done to reveal the binding site of MPO on apolipoprotein A-I of HDL [98]. Furthermore, Carr et al. described MPO : LDL ratio of 3 : 1, whereas Sokolov et al. found a ratio of 1 : 1. The study of MPO-LDL interactions and LDL oxidation by MPO thus remains a challenge for the future.

*Oxidation of LDL by MPO-Dependent Species other than Hypochlorous Acid*. Whereas HOCl is the major oxidant formed by MPO, others might also be produced *in vivo*. Among them, tyrosyl radical, nitrogen dioxide, hypobromous acid, cyanate, and hypothiocyanous acid are often mentioned as potential species that may target LDL. 

Tyrosine is a substrate of MPO, and tyrosyl radical can be produced via the one-electron reaction of the peroxidase cycle. The latter rapidly oxidizes lipids and also forms di-tyrosine residues in proteins, and protein-bound dityrosine residues have been identified in atherosclerotic lesions [99].

Nitration of LDL has been also studied in several works. Recently, Hamilton et al. have shown that LDL nitration leads to unfolded protein and deleterious effects [100]. These authors studied the phenomenon using Sin-1 as a peroxynitrite generator for protein nitration, and MPO has also been used as a catalyzer of nitration [101–103]. However, MPO mediates protein nitration via the formation of nitrogen dioxide (NO_2_^−^) from nitric oxide [104, 105].

MPO can also produce hypobromous and hypothiocyanous acids via the halogenous cycle. The former would be a minor product of MPO activity [49], but several studies have been performed on its reactivity on lipoproteins [106]. The latter work concluded that hypobromous acid attacks both lipid and protein moieties, but it has less deleterious effects than hypochlorous acid. On the other hand, hypothiocyanous formation might be present *in vivo* to a larger extent; however, there are no clear data showing definitive alteration produced by this species [59]. However, cyanate (^−^OCN), a product of decomposition of hypothiocyanous acid, reacts with the terminal amino group of lysine, forming a carbamylated residue, also named homocitrulline. MPO can also use thiocyanate (^−^SCN) and produce ^−^OCN [107]. MPO might therefore indirectly result in protein carbamylation, and this phenomenon was observed in lesions and lipoproteins [107, 108]. It is also worth noting that ^−^SCN concentration may change the efficiency of MPO to produce HOCl [59]. These data illustrate how complex the process of LDL oxidation is *in vivo,* and the latter should be the subject of future experiments.

#### 4.2.3. Localization of LDL Oxidation by Myeloperoxidase

The consensus model of atherogenesis describes the first step of the disease as migration of native LDL from plasma to the subendothelial space where it can be oxidized [17]. However, the mechanism and localization of *in vivo* LDL oxidation is still not fully understood. The model of early LDL oxidation in the circulation is often ruled out by the fact that blood contains lots of antioxidant molecules. It is further thought that the presence of ox-LDL in the plasma is due to backdiffusion from lesions. However, evidence has recently emerged to strengthen the possibility of LDL oxidation in the circulation. Our group has shown that LDL can be oxidized at the surface of activated endothelial cells in the presence of MPO. Circulating MPO is indeed known to adsorb on LDL and also on endothelium where this oxidation process could happen when the cells are activated and NADPH oxidase complex produces O_2_^•−^. To this end, *in vitro* experiments were performed using endothelial cells (Ea.hy926) which were incubated for 24 h in the presence of native LDL, MPO, and angiotensin II, a modulator of O_2_^•−^ production by the NADPH oxidase complex. Mox-LDL production was monitored using a specific Mox-LDL antibody [109] and increased dependently of MPO and LDL concentrations. These data showed that LDL oxidation is possibly not restricted to intima. The plasma level of Mox-LDL is potentially a marker of plasma MPO activity in the field of cardiovascular disease. In this context, we showed that patients exposed to hemodialysis therapy due to kidney failure have higher blood levels of Mox-LDL, and this could be linked to their high cardiovascular risk [110]. 

**Figure 2:**
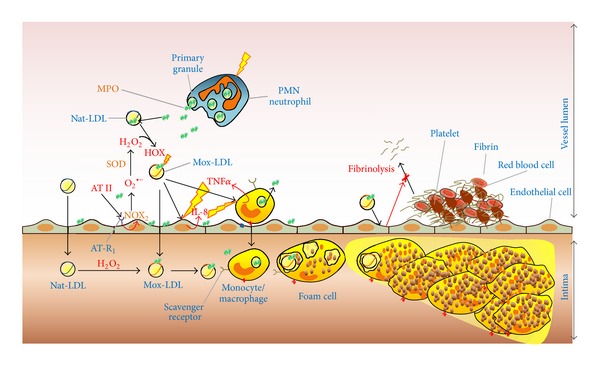
Role of myeloperoxidase and Mox-LDL in triggering inflammation and atherosclerosis plaque formation. Activation of neutrophils and monocytes leads to MPO release in the extracellular space, that is, the circulation. Due to its cationic properties, free MPO rapidly adsorbs at the surface of endothelial cells or native LDL (Nat-LDL). Angiotensin II (AT II) activates endothelial cells via angiotensin receptor 1 (AT-R1), which in turn produces superoxide anion (O_2_^•−^) via the NADPH oxidase complex, (NOX2). O_2_^•−^ is rapidly transformed into hydrogen peroxide (H_2_O_2_) spontaneously or by the enzyme superoxide dismutase (SOD). Nat-LDL can be so directly oxidized by MPO/H_2_O_2_/chloride system in the circulation and form the so-called Mox-LDL. The latter can in turn pass through the endothelium (due to endothelial dysfunction) to the subendothelial space where it will be recognized by macrophages and eliminated. Accumulation of oxidized lipoproteins leads to foam cell formation and lipid accumulation in the subendothelial space. Nat-LDL, can also directly pass through the endothelial wall where they are oxidized by MPO in the subendothelial space. Finally, LDL oxidized by myeloperoxidase (Mox-LDL) activates endothelial cells and induces interleukine-8 (IL-8) secretion by these cells. Mox-LDL effects on monocyte are similar and activate tumor-necrosis factor-*α* (TNF*α*) secretion by these cells. In turn, IL-8 and TNF*α* activate monocytes and endothelial cells, respectively. Mox-LDL also inhibits fibrinolysis process via endothelial cell interaction.

To summarize, Figure 2 illustrates a revised scheme of the LDL oxidation by MPO in atherogenesis taking into account the model of oxidation in the circulation.

## 5. Impacts of Mox-LDL on Inflammation: *In Vitro* Experiments

As mentioned previously in this paper, Mox-LDL is very specific and differs from LDL oxidized by Cu^2+^. In this context, monoclonal antibodies against Mox-LDL were developed for immunochemical studies. In our research group, several antibodies were generated by immunizing mice and collecting and analyzing clones. Four antibodies were specific for Mox-LDL and did not crossreact with Cu-LDL, LDL oxidized by H_2_O_2_, or albumin oxidized by the MPO system. Three of these antibodies recognize the protein moiety of LDL (AG948, EB2E9, and EB2G3), and one (14A2G6) is dependent on the presence of the lipid moiety. Furthermore, the three protein-sensitive antibodies appear to be conformation dependent [109]. These antibodies react with atherosclerotic plaques showing that they can be used for immunohistochemistry studies. Other monoclonal antibodies against Mox-LDL have also been developed by other groups [14].

A large number of transcription factors have been observed to be activated by ox-LDL [111], and many of them have particular impacts on the inflammatory effect of atherosclerosis. In the following paragraphs, we report the major effects observed on monocytes, macrophages, and endothelial cells with a particular interest on Mox-LDL induction.

### 5.1. Effects of Mox-LDL on Monocytes/Macrophages

Atherosclerosis is a complex process involving inflammatory and oxidative stress pathways [112]. Ox-LDL is involved in monocyte/macrophage activation and in the inflammatory response [113]. Monocytes are one of the first cells that reach the site of inflammation such as in nascent atherosclerotic lesions. When activated, this cell type expresses leukocyte adhesion molecules [114], and it also produces ROS and RNS, partly due to MPO activity, and causes the transformation of LDL into a high-uptake form for macrophages [115–117]. Cu-LDL has the capacity to activate monocytes and increases expression of peroxisome proliferator-activated receptor-*γ* (PPAR-*γ*), a regulator of cell proliferation, inflammation, monocyte/macrophage differentiation, and CD36 scavenger receptor expression at the cell surface [118, 119]. In 2005, Westendorf et al. have shown that HOCl-LDL has the same proinflammatory properties *in vitro* [120]. 

As with Cu-LDL [18], HOCl-LDL inhibits lysosomal proteases (e.g., cathepsin B), but the mechanism was identified as dependent on the chloramine content of apoB-100 and oxidized residues that are not present in Cu-LDL [121]. This protease inhibition contributes to lipid accumulation in macrophages and to foam cell transformation.

Furthermore, both Cu- and HOCl-LDL are potent inducers of caspase-dependent apoptosis as shown by Vicca et al. [122] on THP-1 monocytes cell. However, macrophage-differentiated cells seemed to be resistant to apoptosis in these experiments. Nevertheless, this effect is compatible with the idea that macrophages have a prolonged survival and boost atherogenesis. 

Considering the literature of LDL oxidation and cell inflammatory processes, studies of ox-LDL effects on monocytes/macrophages have been mainly performed using Cu-LDL, whereas HOCl-LDL and MPO-LDL are more rarely used. We recently investigated Mox-LDL impacts on a THP-1 cell line and observed an intriguing result [123]. Incubation of Mox-LDL with THP-1 cells during 4 h increased 2-folds the secretion of TNF*α* (a key regulator of the synthesis of acute-phase proteins (i.e., fibrinogen, factor VIII) that are linked to atherogenesis [124]), whereas no increase was detected for native LDL or native and Mox-albumin. These data highlighted the specificity of Mox-LDL as MPO-oxidized proteins did not induce TNF*α* production. TNF*α* is itself an activator of other cells such as endothelial cells where it induces among other things the expression of VCAM-1 [125]. We will return to discuss this activation later in this paper in the context of endothelial cell activation by Mox-LDL.

More recently, macrophage reactivity to ox-LDL was investigated by comparing Mox-LDL and Cu-LDL on RAW264.7 cells [126]. This cell line is usually used for metabolic studies with the advantage of interaction with ox-LDL and to induce foam cell formation [127–130]. Cells were incubated with native and ox-LDL for 48 h before analysis. The first set of experiments highlighted that accumulation of lipids is higher in the presence of Mox-LDL than Cu-LDL (Figure 1 of [126]). The same trend was confirmed with macrophages derived from peripheral blood mononuclear cells (PBMCs) differentiated by macrophage-colony stimulating factor. In a second set of experiments, ROS production was explored by monitoring fluorescence of dichloro-dihydro-fluorescein when RAW264.7 and PBMC-derived macrophages were exposed to ox-LDL. Whereas both Mox- and Cu-LDL significantly increased ROS accumulation in RAW264.7, only Mox-LDL seemed to increase this accumulation in PBMC-derived macrophages (Figure 2 of [126]). 

In order to combat an excess of ROS, cells have developed several mechanisms. In this context, NF-E2-related factor 2 (Nrf2) is a transcription factor which is upregulated when ROS is increased. In redox homeostatic conditions, Nrf2 is inactive and kept in the cytoplasm bound with a Keap1/Rbx1/Cul3 complex, which promotes its ubiquitination and subsequent degradation by the 26S proteasome. When activated, Nrf2 binds DNA at the “antioxidant response element” and regulates the expression of protective genes. The regulatory subunit of glutamate-cysteine ligase (Gclm) and hemeoxygenase-1 (HO-1) are two examples of Nrf2-regulated genes [131]. Glcm is the limiting component of glutathione production, a strong *in vivo* antioxidant, and HO-1 is responsible for hemedegradation in cells, decreasing potential oxidant formation by heme-enzymes. In the same study, Calay et al. [126] showed that Mox-LDL and Cu-LDL result in overexpression of Gclm and HO-1 induction by Nrf2-dependent activation. However, Mox-LDL triggered a stronger response than Cu-LDL. In addition, Trolox, a well-known water-soluble antioxidant able to quench ROS production, was added to experimental batches in order to confirm that Nrf2 was induced by ROS accumulation. Addition of Trolox led to reduced Nrf2 expression. However, while ROS production was totally inhibited by Trolox, Gclm and HO-1 expression was still higher than basal level, suggesting the implication of other pathways in their overexpression. RNA interference approaches targeting Nrf2 gave the same result of partial abolition of Gclm and HO-1 expression and confirmed the hypothesis that transcription factors other than Nrf2 are implicated in the antioxidant response of macrophages.

Differences observed between Cu- and Mox-LDL could be explained by the fact that their ROS induction is mediated by different pathways. ROS production via NADPH oxidase is activated by both ox-LDL, but only Mox-LDL induced the production of ROS by cytosolic phospholipase A2. This was illustrated by quantifying ROS production in the presence of methylarachidonylfluorophosphonate, an inhibitor of cytosolic phospholipase A2. A 43% decrease was observed in ROS production induced by Mox-LDL where no decrease was observed for Cu-LDL.

Interestingly, when Mox-LDL was generated by a stronger MPO-dependent oxidative process, which is capable of extending the oxidation of LDL to the lipid moiety, the ROS production was decreased but remained higher than Cu-LDL induction. These data suggest that lipid peroxidation levels of ox-LDL could be inversely correlated to ROS production in macrophages. 

In summary, Mox-LDL induces ROS production, lipid accumulation, and antioxidant responses in macrophages as with other ox-LDL but by using a different pathway than Cu-LDL. However, Mox-LDL seems to induce a higher responsiveness in monocytes/macrophages than ox-LDL and might be more atherogenic. 

### 5.2. Effects of Mox-LDL on Endothelial Cells

Endothelial dysfunction is potentially the first event of atherosclerosis development. It is still not totally understood why this occurs and when these lesions start, but evidence has been claimed many times. Endothelial permeability is increased at the site of lesions and favors LDL penetration into the artery wall. Furthermore, ox-LDL mediates endothelial dysfunction and is considered as a key event in the initiation of arterial lesions [132].

Endothelial cells also express scavenger receptor (SR) at their surface and can interact with ox-LDL. However, the main SR expressed on endothelial cells is LOX-1, the specific lectin-like endothelial receptor for ox-LDL. However, CD36 and SR-B1 have also been localized at the endothelial cell surface [133]. HOCl-LDL is internalized by CD36 and SR-B1 [134], while the receptor(s) which recognize(s) Mox-LDL and enable(s) endocytosis remain(s) to be documented for monocytes, macrophages, and endothelial cells. The presence of scavenger receptors at the surface of endothelial cells could lead to endocytosis of Mox-LDL. However, endothelial cells are not able to accumulate lipids when incubated with ox-LDL. Nevertheless, Mox-LDL activates endothelial cells, as well as monocytes/macrophages.

Cu-LDL but not native LDL is able to induce interleukin-8 (IL-8) production by endothelial cells [135]. IL-8 belongs to the C-X-C subgroup of chemokines and is a multifunctional cytokine involved in numerous biological processes including atherosclerosis. IL-8 acts as a chemoattractant to inflammatory cells and also to smooth muscle cells and is involved in the migration of the latter in the intima. Furthermore, IL-8 activates monocytes and/or macrophages and up-regulates their production of TNF*α*. HOCl-LDL induction of IL-8 was also demonstrated but only in monocytes [136].

In order to assess whether Mox-LDL is also an inducer of IL-8 production by endothelial cells, our group performed an experiment where Mox-LDL was incubated with EAhy926 endothelial cells for 48 h [123]. This cell line is a reliable model for studying vascular inflammation, leukocyte-endothelial interactions, and metabolic impacts of ox-LDL [135]. IL-8 was measured in the supernatant, and a dose-dependent response was observed. No response was detected with native LDL and native or Mox-albumin (control experiments). The specificity of Mox-LDL was also confirmed by the absence of a Mox-albumin effect. 

With the capability of Mox-LDL to trigger both monocytes and endothelial cells, it appeared that a cycle could be present. Indeed, Mox-LDL activated both monocytes and endothelial cells which secrete TNF*α* and IL-8 respectively. The latter activates endothelial cells and monocytes/macrophages leading (i) to ROS production, (ii) to MPO release in the extracellular matrix, and (iii) so potentially to new Mox-LDL.

### 5.3. Mox-LDL and Fibrinolysis

Coagulation and fibrinolysis are continuously in balance at the surface of the endothelial cell wall. These cells contribute to fibrinolysis by secreting tissue-plasminogen activator (t-PA), urokinase-plasminogen activator (u-PA), and plasminogen activator inhibitor-1 (PAI-1), three fibrinolysis regulators, or by expressing specific receptors which bind these fibrinolysis factors [137]. Enhancement of fibrin generation gives a prothrombotic environment on the endothelial cell surface, and fibrin induces the production of IL-8 by endothelial cells. In this context, a dysfunctional fibrinolysis process was reported to be a factor in atherogenesis, complementary to ox-LDL [138]. This was first mentioned as a key factor in 1998 by Sueishi et al. [139] and Mayerl et al. [140], and a recent clinical study from our group further confirmed it [141]. However, until 2012, the interplay between Mox-LDL (and more generally ox-LDL), endothelial cells, and fibrinolysis had not been investigated [142]. Our group documented this intriguing subject with the aid of a device which allows us to monitor fibrinolysis in real time [143]. Briefly, fibrin formation and degradation occurs in adapted circular microcuvettes. To monitor the effect of endothelial cells (EA.hy926) on fibrinolysis taking place at their surface, cells were immobilized on collagen-coated membranes, fixed to the bottom of glass circular microcuvettes, and grown to confluence. The microcuvettes were inserted in the experimental apparatus at 37°C, the euglobuin fraction was added and clot formation started by addition of thrombin. TNF*α* was used as a positive control as it is known to have antifibrinolytic activity. To test the system, a 24 h TNF*α* treatment of endothelial cells was performed and effectively showed an increase in fibrinolysis time. Monitoring of native and Mox-LDL showed that Mox-LDL at concentrations of 10 and 50 *μ*g/mL also increased the time of fibrinolysis unlike native lipoprotein, confirming again that Mox-LDL has a physiopathological effect on atherogenesis. Nevertheless, higher concentrations of Mox-LDL (100 *μ*g/mL) showed a decreased effect. 

PAI-1, t-PA, annexin II (a t-PA receptor), and uPAR secretion were also analyzed in this study, but there was no effect of native or Mox-LDL. However, as TNF*α* increased t-PA and PAI-1 in smooth muscle cells, it is suggested that other pathways/factors are involved in fibrinolysis modulation. This raises the question whether the receptor and signal transduction pathways activated by Mox-LDL and TNF*α* are different. With a view to clarify the underlying bio-molecular mechanism, scavenger receptor interactions with Mox-LDL were investigated. Previous data described LOX-1 binding to ox-LDL and mediating effects in endothelial cells. However, our first investigations by neutralization of this receptor using antibodies did not impact IL-8 production induced by Mox-LDL, disproving this pathway [142]. Hence, future research on scavenger receptor is required to extend the understanding of Mox-LDL effects on endothelial cells. 

In summary, Mox-LDL disturbs fibrinolysis but in a different pathway than the t-PA- and PAI-1 dependent pathways. Future investigations are needed to solve the mechanism by which Mox-LDL is involved in this pathological process.

### 5.4. Summary of *In Vitro* Effects of Mox-LDL

It appears that Mox-LDL plays a crucial role in lipid accumulation in macrophages/foam cells and also in the whole proinflammatory process linked to atherosclerosis lesion development. Figure 2 summarizes the different aspects and effects of MPO/Mox-LDL in the circulation/intima including effects on endothelial cells, monocytes/macrophages, and fibrinolysis. Mox-LDL generates a vicious circle effect, prevents resolution of the nascent lesion, triggers oxidative stress and lipid accumulation in the subendothelial space, and inhibits normal fibrinolysis. 

## 6. Clinical Aspects of Mox-LDL

### 6.1. Introduction

For a long time, ox-LDL and MPO have been accepted as cardiovascular risk factors and have been largely documented in the literature by *in vivo* experiments or clinical studies [17, 63, 144–146]. However, only a small number of studies have investigated LDL modified by the MPO/H_2_O_2_/chloride system (Mox-LDL). Our group has contributed to this, and we observed that Mox-LDL is present in atherosclerotic lesions [109]. We have already discussed numerous Mox-LDL effects in atherosclerosis plaque formation in this paper and this subject is not further discussed here. Nevertheless, cardiovascular diseases are linked, notably in relation to atherosclerosis development, to several pathologies such as kidney failure/end stage renal disease, sleep restriction, erectile dysfunction, or chronic obstructive pulmonary disease. Evidences have been growing for Mox-LDL implications in these pathologies, and they are summarized in the following paragraphs. 

### 6.2. Mox-LDL, Kidney Failure, and Hemodialysis

Kidney failure and subsequent uremia has been linked to chronic inflammation and cardiovascular disease [147]. It has also been proposed that hemodialysis triggers inflammation as a result of exposure of blood to the bioincompatible system stimulating monocyte and macrophage cells [148, 149]. These processes induce proinflammatory oxidative stress responses, and MPO has been implicated in the development of cardiovascular and chronic kidney diseases [88, 150, 151]. MPO also directly targets kidneys through HOCl production [152, 153]. 

Wu et al. reported [154] that MPO concentration could serve as a marker of oxidative stress during hemodialysis, and Himmelfarb et al. [155] showed that MPO concentration increases during hemodialysis sessions. We also recently contributed to MPO investigations in the context of hemodialysis therapy [156]. In this paper, MPO activity was monitored using the SIEFED (specific immunoextraction followed by enzymatic detection) method developed previously by Franck et al. [157]. We further developed a total protein hydrolysis method assisted by microwave and coupled to LC-MS analyses devoted to the monitoring of protein-bound 3-chlorotyrosine and homocitrulline. In this work, it was observed in 15 patients that an increase of plasma MPO, levels during the hemodialysis, is accompanied (i) by a direct increase of MPO activity and, more interestingly, (ii) by a direct MPO-dependent oxidation of plasma proteins. 

Previously, we had shown that Mox-LDL levels in blood are increased during hemodialysis [110]. Together with the recent results, these data highlight that MPO induces direct protein oxidation and potentially targets LDL. Indeed, MPO avidly interacts and adsorbs at the surface of LDL and triggers apoB-100 oxidation with an impact on the cardiovascular risk of the patients [156]. Furthermore, these data strengthen the model of the MPO-dependent oxidation of LDL in the circulation as a contributive mechanism of atherosclerosis. The latter process is also illustrated in Figure 2.

### 6.3. Mox-LDL and Sleep Restriction

Nowadays, people are more and more sleep deprived due to work pressure and requirement (i.e., shift schedules or extension of hours), family demands, or our 24/7-week lifestyle [158]. According to the 2009 National Sleep Foundation Survey, 20% of Americans sleep less than 6 h per night during the week [159]. This modification of sleep duration is not of minor consequence to our health. Sleep deprivation is harmful and can lead to problems in metabolism [160], immune [161], or cardiovascular systems [162, 163]. 

Studying sleep-deprived individuals, van Leeuwen et al. have associated the increase of proinflammatory molecules IL-17, C-reactive protein (CRP), IL-1*β*, and IL-6 with cardiovascular risk [164]. Furthermore, total or severe sleep restriction alters blood cell counts with a particular increasing effect on neutrophils and granulocytes [165, 166]. Neutrophil count has even been proposed as a marker of immune recovery function of sleep [167]. An increase in MPO plasma levels was also detected after acute sleep restriction [166].

In this context, MPO and Mox-LDL levels were recently studied, together with inflammatory markers in 9 mid-twenties men for 11 consecutive days (3 baseline nights followed by 5 restricted-sleep nights (max. 5 hours of sleep) and then 3 recovery-sleep nights) [168]. Results showed that MPO was not increased during the sleep restriction but rose during the first night of recovery sleep. Whereas MPO levels peaked during the first recovery night, Mox-LDL levels were significantly higher during the first and third nights of sleep restriction but not at the recovery period. Mox-LDL/apoB-100 ratio, which expresses the fraction of modified lipoproteins in the total pool, was also statistically increased during the first night of sleep restriction. Speculating on the reason for this temporal discordance between Mox-LDL and MPO levels in blood, it appears from the literature that catecholamines are increased during sleep restriction [169] and can activate the NADPH oxidase complex at the surface of endothelial cells [170]. As a result, the O_2_^−•^ formed could be converted into H_2_O_2_ and react with MPO to form Mox-LDL. 

In summary, these data show that the recovery process after sleep restriction is linked to modifications of levels of cardiovascular risk biomarkers in blood. However, future experiments are required to help understand the impact of sleep restriction on human health. Studies including more male and female individuals are also needed but are unfortunately difficult to set up and standardize. 

### 6.4. Mox-LDL and Nonalcoholic Fatty Liver Disease

Nonalcoholic fatty liver disease (NAFLD) includes different liver disorders such as steatosis, steatohepatitis, cirrhosis, and advanced fibrosis. Furthermore, NAFLD has been associated with a high risk of cardiovascular disease (CVD) [171], obstructive sleep apnea [172], or colorectal cancer [173]. MPO has been involved in the progression of non-alcoholic steatohepatitis where neutrophil accumulation is a key component of the inflammatory process. Moreover, it has been shown that NAFLD is associated with increased levels of nitrated proteins that might partly come from MPO activity [174]. More recently, Rensen et al. reported that MPO deficiency decreases hepatic cholesterol accumulation and inflammation in mice that do not express the LDL-receptor (LDLR^−/−^ mice) and that were fed with a high-fat diet, [175]. In these experiments, the authors also observed that, after 3 weeks of high-fat diet, MPO levels were increased in the liver of hyperlipidemic mice that expressed MPO. Furthermore, MPO activity in mouse liver was investigated by monitoring nitrotyrosine levels. The latter product is indeed, at least partially, generated by MPO during the inflammation process. These data demonstrate the important role of MPO in NAFLD, and this might be linked to the oxidation of lipoproteins and particularly of LDL by MPO.

### 6.5. Mox-LDL and Chronic Obstructive Pulmonary Disease

Patients with chronic obstructive pulmonary disease (COPD) have increased systemic inflammation, increased endothelial dysfunction, and changes in the oxidant/antioxidant ratio. Furthermore, these patients are at a high risk of cardiovascular disease, and 22%–50% of them will die of cardiovascular conditions [176–178]. Long-term oxygen therapy has been observed to prolong survival in hypoxemic COPD patients, but the mechanisms are not completely understood.

In this context, we hypothesized that oxygen therapy could alter systemic inflammation and oxidative stress. As a consequence, several markers, including neutrophils, Mox-LDL, and IL-8, were monitored in 11 patients before starting, after one week and after one month of oxygen therapy [179]. Neutrophils, IL-8, and Mox-LDL were all significantly decreased after one month of oxygen therapy. These data showed that oxygen breathing is favorable to reduce the oxidative stress and inflammatory state in hypoxemic patients with COPD. 

At this point only, speculation could explain these observed decreases. In this way, chronic hypoxia is associated with raised sympathetic activity, activation of the renin-angiotensin system, and production of catecholamines [180]. By activating the NADPH oxidase complex, catecholamines can induce Mox-LDL formation in the circulation. Conversely, when oxygen therapy is provided, catecholamine formation is reduced, which could decrease Mox-LDL formation. Reduction of the sympathetic activity might also decrease neutrophil counts in blood and act on MPO level. Finally, IL-8 released by endothelial cells is also induced by Mox-LDL *in vitro* and suggests that Mox-LDL decrease could partly be explained by the reduction in IL-8 secretion. 

### 6.6. Mox-LDL and Erectile Dysfunction

Erectile dysfunction (ED) is a vascular disorder. Indeed, erection and detumescence of the penis are hemodynamic events controlled by the relaxation and contraction, respectively, of arterial and intracavernous smooth muscle cells. Erectile function is also subject to endothelial cells and their ability to release nitric oxide (NO^•^). NO^•^ is indeed the main neurotransmitter involved in erection and reacts with the enzyme guanylate cyclase that increases cyclic guanosine monophosphate and produces a cascade of events at the intracellular level. This process results in a loss of contractile tone of smooth muscle cells [181, 182]. 

Clinical studies have identified a link between hypercholesterolemia, atherosclerosis, and erectile dysfunction. ED has been even described as a preliminary event of future major cardiovascular outcomes and as a predictor of coronary heart disease [183–186]. Furthermore, it was reported that HOCl-LDL inhibits NO^•^ synthesis in endothelium [187], and we previously reported effects of Mox-LDL on endothelial cells ([123] and see above: effects of Mox-LDL on endothelial cells). 

On this basis, our group has performed an immunohistochemical approach to study the presence of Mox-LDL in penile tissues in patients suffering from ED [188]. Intracavernous tissue was taken from 8 patients undergoing penile implant surgery, and an antibody against Mox-LDL [109] was used to reveal the presence of MPO-dependent oxidized LDL. Among these patients, 7 were known to have vascular ED, and one patient had ED due to neurologic lesions after radical prostatectomy. In the 7 patients with vascular ED, the presence of Mox-LDL was observed, whereas no Mox-LDL was observed in the patient with ED due to prostatectomy. The latter was a good negative control and these data confirmed that, when ED is due to vascular dysfunction, LDL is oxidized and this phenomenon could trigger ED development. 

In addition, careful observation of stained slides revealed that Mox-LDL is restricted to the endothelium and subendothelial space in artery, but, conversely, they are deeply diffused and intermingled between the smooth muscle fibers in the intracavernous tissues. Furthermore, Mox-LDL staining revealed the presence of Mox-LDL in the cytoplasm of endothelial cells. This confirms the endocytosis of these lipoproteins into cells. 

As cyclic guanosine monophosphate (cGMP) is a key mediator in the NO^•^-dependent pathway of erectile function, it was hypothesized that cGMP levels could be influenced by the presence of Mox-LDL. We showed *in vitro* that a 48 h incubation of Mox-LDL with EA.hy926 endothelial cells induces a decrease of the level of cGMP when compared with control and native LDL [189]. In the cavernosum tissue, cGMP is naturally hydrolyzed by phosphodiesterase 5 (PDE5). Inhibition of this enzyme is considered a first-line therapy for patients with ED and helps maintain higher levels of cGMP [190–192]. Thus, Mox-LDL presence in penile tissue could be an explanation for the resistance of patients to PDE5 inhibitor therapy [189]. Conversely, PDE5 inhibitors were tested to know whether they protect or not against the proinflammatory effects of Mox-LDL on endothelial cells. For this purpose, EA.hy926 endothelial cells were incubated with Mox-LDL and available PDE5 inhibitors (sildenafil, vardenafil and tadalafil), and IL-8 production was then measured [193]. Only one of the PDE5 inhibitors (tadalafil) was shown to have a beneficial effect *in vitro* by significantly decreasing IL-8 production compared with the other two inhibitors. A complementary effect on endothelial cells (in addition to the relaxation) could be also produced by tadalafil. This could potentially be an interesting effect that could be considered in the future when a physician implements a chronic treatment for ED. However, clinical data are required to confirm the *in vitro* experiments before drawing further conclusions.

## 7. Concluding Remarks and Future Perspectives

In summary and conclusion, *in vitro* experiments and clinical studies support a key role of MPO-dependent oxidized LDL in the process of atherosclerosis and cardiovascular linked diseases. This specifically modified lipoprotein induces proinflammatory effects by stimulating TNF*α* and IL-8 secretion in monocytes and endothelial cells respectively. In turn, TNF*α* and IL-8 activate endothelial cells and monocytes, respectively, and promote MPO and ROS release leading to new Mox-LDL formation. Furthermore, Mox-LDL has exhibited inhibitory effects on fibrinolysis, a key process in the release of fibrin. 

*In vivo*, Mox-LDL is present in atherosclerotic lesions, and its level is increased in the circulation of patients with high cardiovascular risk, such as those with kidney failure and those undergoing hemodialysis. The presence of Mox-LDL was also revealed in patients suffering from vascular erectile dysfunction, disease linked to atherosclerosis and endothelial dysfunction. In addition, increased blood levels of Mox-LDL have also been observed during sleep restriction. Mox-LDL is thus a potentially good marker of cardiovascular disorders and/or cardiovascular risk. 

In contrast to other types of oxidation (e.g., by copper), MPO-dependent oxidation of LDL primarily targets the protein moiety of LDL, namely, apolipoprotein B-100. Furthermore, MPO oxidation is thought of as a more physiopathological model of LDL oxidation than that involving copper. As Mox-LDL induces much more ROS production and lipid accumulation in macrophages than Cu-LDL, Mox-LDL in particular should be considered more in biological experiments and clinical studies. 

Challenges thus remain for the future, and researchers should keep working on the impact of MPO and Mox-LDL on human health. As an example, the exact receptors that enable Mox-LDL endocytosis in macrophages/monocytes and endothelial cells should be further studied. Last but not least, it has been observed that MPO specifically oxidizes apoB-100, but the exact binding sites of MPO at the surface of apoB-100 remain to be further described, as well as the residues oxidized on apoB-100. Increased understanding of the impact of Mox-LDL on the induction of proinflammatory and oxidative stress processes is also of major importance for the future. 

## Abbreviations

ApoB-100: Apolipoprotein B-100
Cu-LDL: Low-density lipoprotein modified by reagent Cu^2+^
ED: Erectile dysfunction
H_2_O_2_: Hydrogen peroxide
HOCl/OCl^−^: Hypochlorous acid/hypochlorite
HOCl-LDL: Low-density lipoprotein modified by reagent HOCl
IL: Interleukin
LDL: Low-density lipoprotein
M-CSF: Macrophage colony stimulating factor
Mox-albumin: Albumin modified by the MPO-H_2_O_2_-chloride system
Mox-LDL: LDL modified by the MPO-H_2_O_2_-chloride system
MPO: Myeloperoxidase
MS: Mass spectrometry
NADPHox: Nicotinamide adenine dinucleotide phosphate oxidase
Ox-LDL: Oxidized LDL
PTM: post-translational modification
RNS: reactive nitrogen species
ROS: reactive oxygen species
TNF*α*: tumor-necrosis factor-*α*.
